# Supplementary material for: Recommended distances for physical distancing during COVID-19 pandemics reveal cultural connections between countries
Source: PLoS One. 2023 Dec 15;18(12):e0289998. doi: 10.1371/journal.pone.0289998 (PMC10723704; doi:10.1371/journal.pone.0289998)
Supplement: S1 Fig — Boxplot of the estimated effective reproduction rate in May 8th, 2020 (A) and in Aug 1st, 2020 (B) plotted against the recommended distance during COVID-19 pandemic. Gaussian generalized linear model of the estimated effective reproduction rate in May 8th, 2020 (C) and in Aug 1st, 2020 (D) according to the recommended minimal distance. (PDF) [file pone.0289998.s001.pdf]

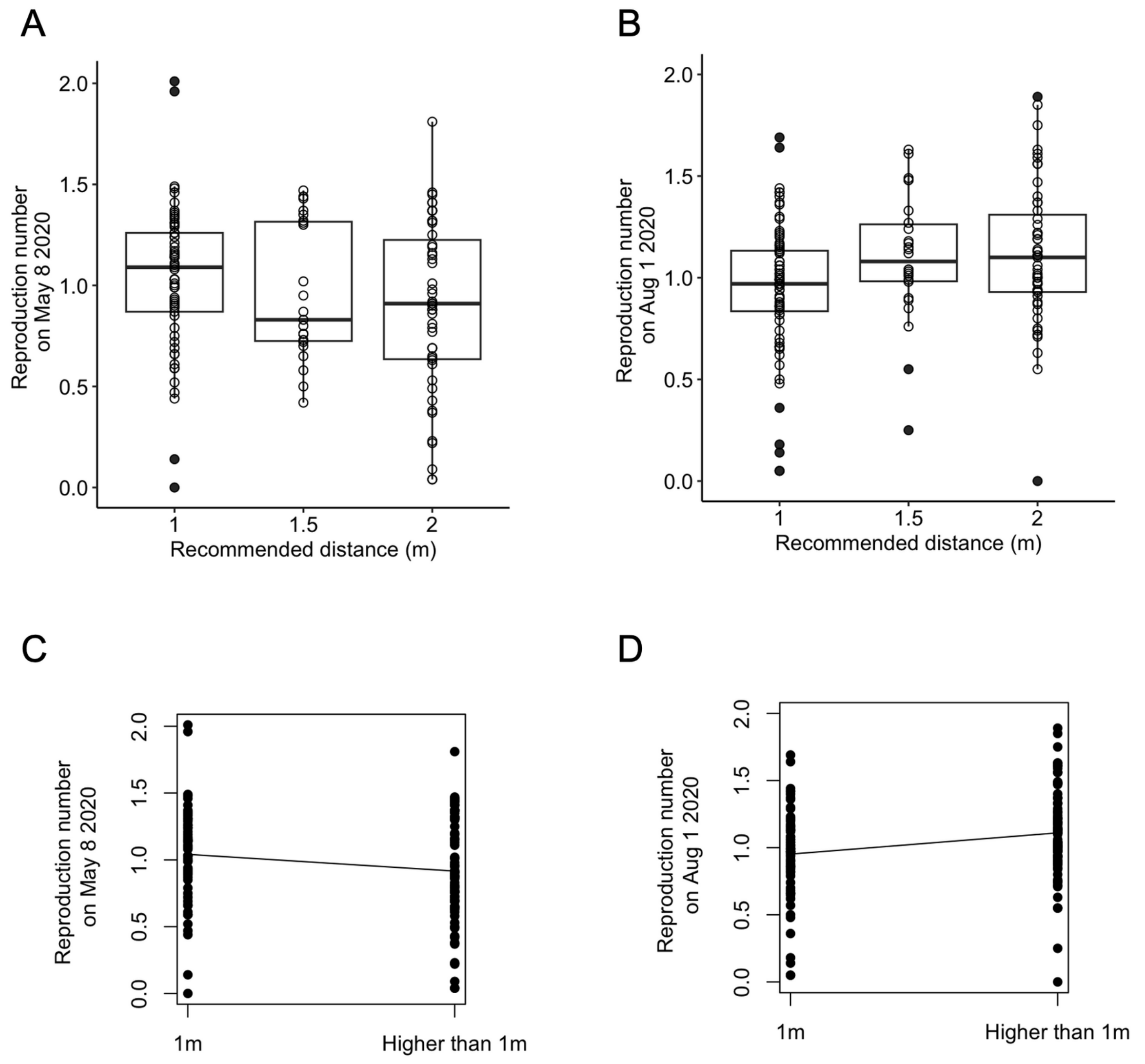

Fig S1. Boxplot & Generalized linear model of the recommended distance and the effective reproduction number R_t (model using Ritchie et al. data). Boxplot of the estimated effective reproduction rate in May 8th, 2020 (A) and in Aug 1st, 2020 (B) plotted against the recommended distance during COVID-19 pandemic. Gaussian generalized linear model of the estimated effective reproduction rate in May 8th, 2020 (C) and in Aug 1st, 2020 (D) according to the recommended minimal distance.
