# Supplementary material for: Recommended distances for physical distancing during COVID-19 pandemics reveal cultural connections between countries
Source: PLoS One. 2023 Dec 15;18(12):e0289998. doi: 10.1371/journal.pone.0289998 (PMC10723704; doi:10.1371/journal.pone.0289998)
Supplement: S2 Fig — Each point represents one country. Countries with a recommended distance of 1–2 m were considered as 1.5m in this graph. The y-axis indicates the social (A), personal (B) and intimate (C) interpersonal distances reported in [13]. (PDF) [file pone.0289998.s002.pdf]

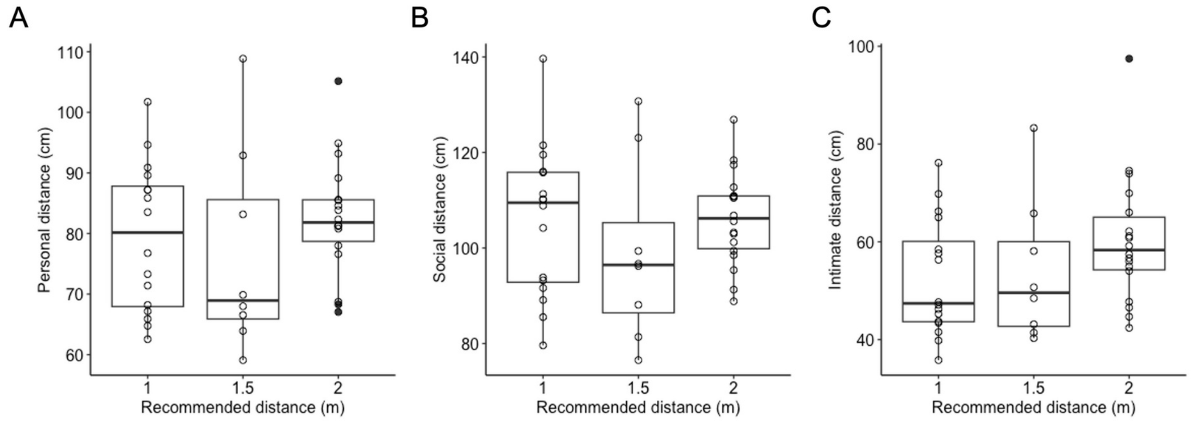

Fig S2. Interpersonal distance of 42 countries plotted against the recommended distance during COVID-19 pandemic. Each point represents one country. Countries with a recommended distance of 1-2 m were considered as 1.5m in this graph. The y-axis indicates the social (A), personal (B) and intimate (C) interpersonal distances reported in [13].
