# Supplementary material for: Recommended distances for physical distancing during COVID-19 pandemics reveal cultural connections between countries
Source: PLoS One. 2023 Dec 15;18(12):e0289998. doi: 10.1371/journal.pone.0289998 (PMC10723704; doi:10.1371/journal.pone.0289998)
Supplement: S3 Fig — (A) Boxplot of population density according to the recommended distance. Each point represents one country. Countries with a recommended distance of 1–2 m were considered as 1.5m in this graph. (B) The y-axis indicates the probability to be at the highest recommended distance (higher than 1m) from the binomial generalized linear model. Points represent data for 174 countries. (PDF) [file pone.0289998.s003.pdf]

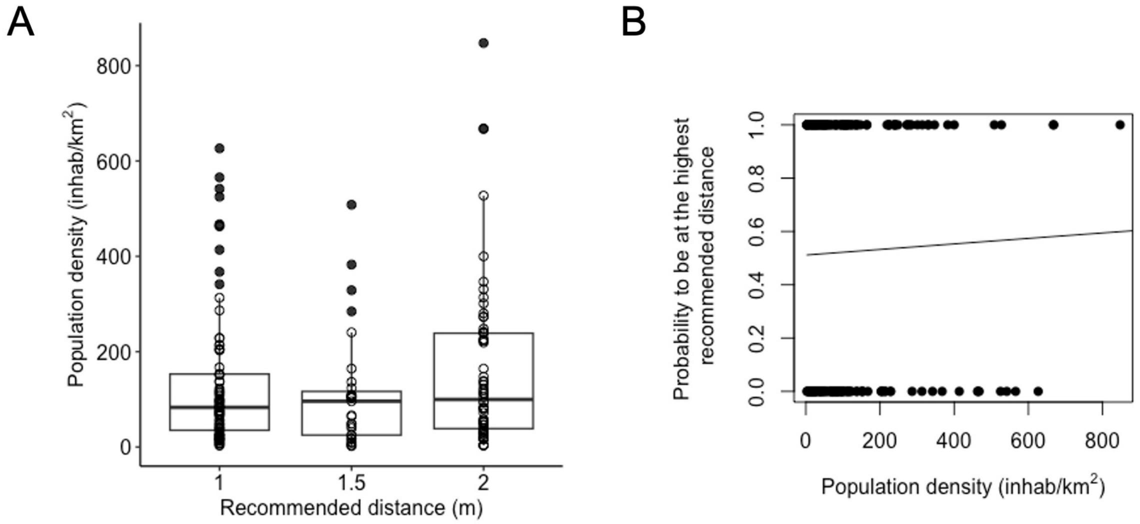

Fig S3. Recommended distances and population density. (A) Boxplot of population density according to the recommended distance. Each point represents one country. Countries with a recommended distance of 1-2 m were considered as 1.5m in this graph. (B) The y-axis indicates the probability to be at the highest recommended distance (higher than 1m) from the binomial generalized linear model. Points represent data for 174 countries.
