# Supplementary material for: Recommended distances for physical distancing during COVID-19 pandemics reveal cultural connections between countries
Source: PLoS One. 2023 Dec 15;18(12):e0289998. doi: 10.1371/journal.pone.0289998 (PMC10723704; doi:10.1371/journal.pone.0289998)
Supplement: S4 Fig — (A) Average recommended distance for civil law (n = 75), common law (n = 21), mixed (n = 85) countries. (B) Average recommended distance for countries previously colonized by Spain (n = 17), France (n = 26), Great Britain (n = 60), Portugal (n = 7), Russia (n = 14) and Turkey (n = 9). (C) Average recommended distance for countries previously exposed (n = 27) or not (n = 157) to SARS-CoV-1. (D) Average recommended distance for countries using CFA Franc (n = 14), Eastern Caribbean Dollar (n = 6), Euro (n = 19), United States Dollar (n = 8). Bars indicate the standard errors. **: p<0.05, ***:p<0.01, 1-way ANOVA followed by Tukey’s multiple comparisons test. (PDF) [file pone.0289998.s004.pdf]

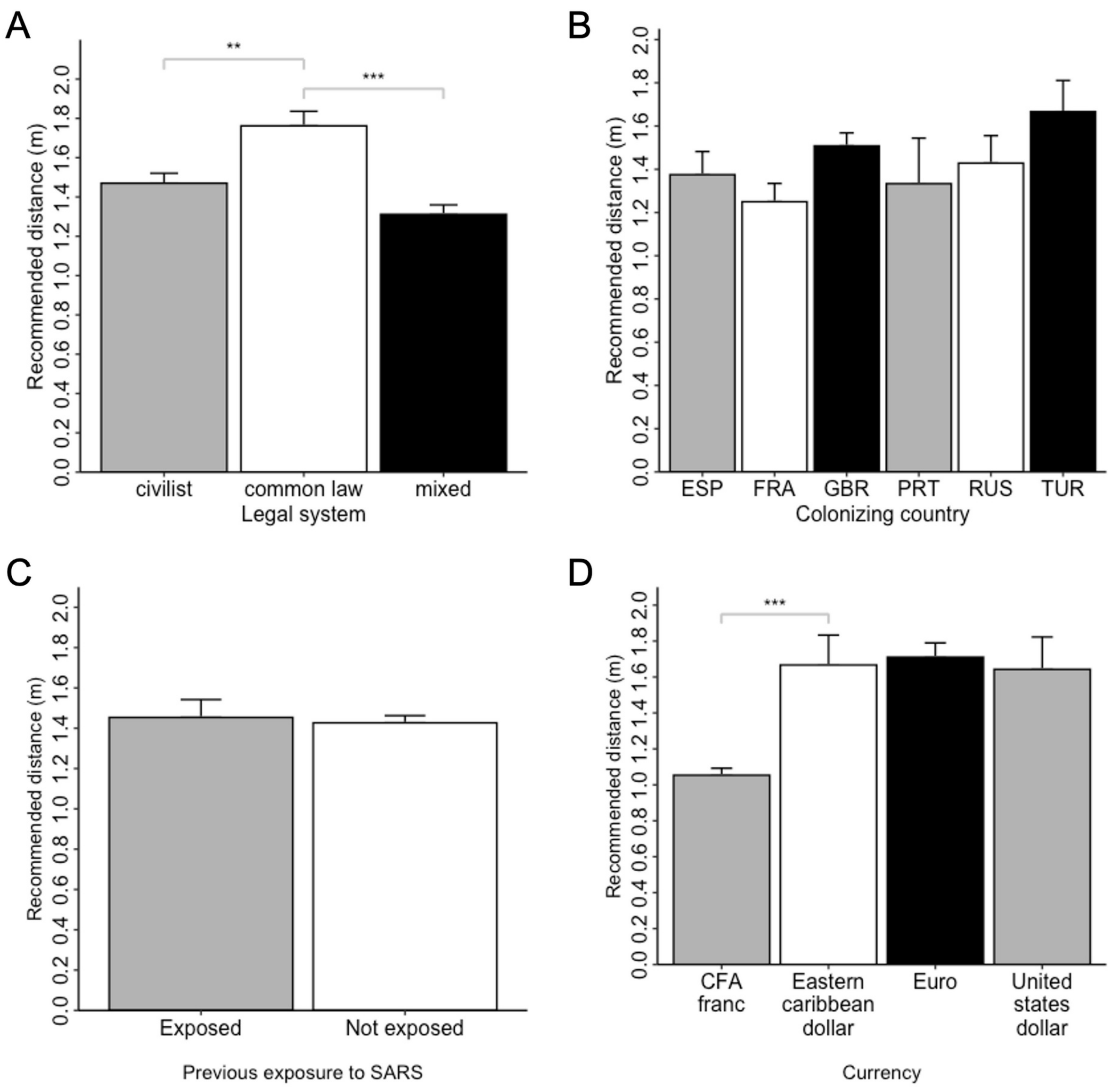

Fig S4. Recommended distances with respect to cultural parameters. (A) Average recommended distance for civil law (n=75), common law (n=21), mixed (n=85) countries. (B) Average recommended distance for countries previously colonized by Spain (n=17), France (n=26), Great Britain (n=60), Portugal (n=7), Russia (n=14) and Turkey (n=9). (C) Average recommended distance for countries previously exposed (n=27) or not (n=157) to SARS-CoV-1. (D) Average recommended distance for countries using CFA Franc (n=14), Eastern Caribbean Dollar (n=6), Euro (n=19), United States Dollar (n=8). Bars indicate the standard errors. **: $p<0.05$, ***:$p<0.01$, 1-way ANOVA followed by Tukey's multiple comparisons test.
